# Supplementary material for: Magnitude and correlates of alcohol use disorder in south Gondar zone, northwest Ethiopia: A community based cross-sectional study
Source: PLoS One. 2021 Sep 30;16(9):e0257804. doi: 10.1371/journal.pone.0257804 (PMC8483395; doi:10.1371/journal.pone.0257804)
Supplement: S1 File — (DOCX) [file pone.0257804.s001.docx]

## Annex II: English Version Questionnaire

Code number: ______________________________

Data collector’s name & signature: Name: ___________________ Signature_________

Supervisor’s name & signature: Name: ___________________Signature__________

**Part 1. socio-demographic information**

| No | Questionnaire | Alternative response | | Coding |
| --- | --- | --- | --- | --- |
| Q-101 | How old are you? | Age in years………. | |  |
| Q-102 | Sex | 1. Male 2. female | |  |
| Q-103 | What is your religion? | 1. Orthodox  2. Muslin  3. Protestant  4.Catholic  5. Others…………. | |  |
| Q-104 | What is your marital status? | 1.Single  2. Married and living together  3. Married but not living together  4. Divorced  5. Widowed | |  |
| Q-105 | What is your ethnicity? | | 1. Amhara 2. Other |  |
| Q-106 | What is the level of your education? | 1.unable to read and write  2.grade 1-8^th^  3.grade9-12^th^  4.diploma and above | |  |
| Q-107 | What is your job? | 1. Governmental employed 2. Merchant 3. Farmer 4. Student 5. Day laborer 6. House wife 7. Non-employed | |  |
| Q-108 | With whom are you living now? | 1. With family 2. Alone | |  |
| Q-109 | What is your place of residence? | 1. Rural 2. Urban | |  |
|  | | | | |

**Part 2:** **AUDIT questionnaire**

Read questions as written. Record answers carefully. Begin the AUDIT by saying “Now I am going to ask you some questions about your use of alcoholic beverages during this past year.” Explain what is meant by “alcoholic beverages” by using local examples of beer, wine, vodka, Tella, Araki, Teje, etc.

| s.no | Question | 0 | 1 | 2 | 3 | 4 | Given point |
| --- | --- | --- | --- | --- | --- | --- | --- |
| 201 | How often do you have a drink containing alcohol? | Never | Monthly or less | 2-4 times a month | 2-3 times a week | 4ormore times a week |  |
| 202 | How many standard drinks containing alcohol do you have on a typical day when drinking? | 1 or 2 | 3 or 4 | 5 or 6 | 7 to 9 | 10 or more |  |
| 203 | How often do you have six or more drinks on one occasion? | Never | Less than monthly | Monthly | Weekly | Daily or almost daily |  |
| 204 | During the past year, how often have you found that you were not able to stop drinking once you had started? | Never | Less than monthly | Monthly | Weekly | Daily or almost daily |  |
| 205 | During the past year, how often have you failed to do what was normally expected of you because of drinking? | Never | Less than monthly | Monthly | Weekly | Daily or almost daily |  |
| 206 | During the past year, how often have you needed a drink in the morning to get yourself going after a heavy drinking session? | Never | Less than monthly | Monthly | Weekly | Daily or almost daily |  |
| 207 | During the past year, how often have you had a feeling of guilt or remorse after drinking? | Never | Less than monthly | Monthly | Weekly | Daily or almost daily |  |
| 208 | During the past year, have you been unable to remember what happened the night before because you had been drinking? | Never | Less than monthly | Monthly | Weekly | Daily or almost daily |  |
| 209 | Have you or someone else been injured as a result of your drinking? | No |  | Yes, but not in the past year |  | Yes, during the past year |  |
| 210 | Has a relative or friend, doctor or other health worker been concerned about your drinking or suggested you cut down? | No |  | Yes, but not in the past year |  | Yes, during the past year |  |
|  | TOTAL |  |  |  |  |  |  |

**Part 3**: I am going to ask you some questions to assess substance use in your life time and in recent times other than medications which didn’t prescribe by health care professionals (Use ”√” to indicate your answer)

| 301 | In your life, which of the following substances have you ever used? (non-medical use only) | YES | NO |
| --- | --- | --- | --- |
|  | A. Tobacco products(cigarettes) |  |  |
|  | B. Amphetamine type stimulants(khat) |  |  |
|  | C. cannabis |  |  |
| 302 | Currently and in the last three months, which of the following substances have you ever used? (non-medical use only) |  |  |
|  | A. Tobacco products(cigarettes) |  |  |
|  | B. Amphetamine type stimulants (khat) |  |  |
|  | C. cannabis |  |  |

**Part 4**: CLINICAL FACTORS

401. Have you ever diagnosed with any mental illness?

1. yes 2. no

402. Did you have any family history of psychiatric illness?

1. yes 2. no

403. Did you have any diagnosed medical, neurological, or surgical illness?

1. yes 2. no

**Part:5 Social Phobia Inventory (SPIN)**

Circle the number that best describes how much the following problems have bothered you during the past week:

| **S.N.** | **Question** | **Not at all** | **A little** | **Moderately** | **A lot** | **Extremely** |
| --- | --- | --- | --- | --- | --- | --- |
| 501 | I am afraid of people in authority | 0 | 1 | 2 | 3 | 4 |
| 502 | I am bothered by blushing in front of people | 0 | 1 | 2 | 3 | 4 |
| 503 | Parties and social events scare me | 0 | 1 | 2 | 3 | 4 |
| 504 | I avoid talking to people I don't know | 0 | 1 | 2 | 3 | 4 |
| 505 | Being criticized scares me a lot | 0 | 1 | 2 | 3 | 4 |
| 506 | Fear of embarrassment causes me to avoid doing things or speaking to people | 0 | 1 | 2 | 3 | 4 |
| 507 | Sweating in front of people causes me distress | 0 | 1 | 2 | 3 | 4 |
| 508 | I avoid going to parties | 0 | 1 | 2 | 3 | 4 |
| 509 | I avoid activities in which I am the center of attention | 0 | 1 | 2 | 3 | 4 |
| 510 | Talking to strangers scares me | 0 | 1 | 2 | 3 | 4 |
| 511 | I avoid having to give speeches | 0 | 1 | 2 | 3 | 4 |
| 512 | I would do anything to avoid being criticized | 0 | 1 | 2 | 3 | 4 |
| 513 | Heart palpitations bother me when I am around people | 0 | 1 | 2 | 3 | 4 |
| 514 | I am afraid of doing things when people might be watching | 0 | 1 | 2 | 3 | 4 |
| 515 | Being embarrassed or looking stupid are my worst fears | 0 | 1 | 2 | 3 | 4 |
| 516 | I avoid speaking to anyone in authority | 0 | 1 | 2 | 3 | 4 |
| 517 | Trembling or shaking in front of others is distressing to me. | 0 | 1 | 2 | 3 | 4 |

**Part 6: PATIENT HEALTH QUESTIONNARIE (PHQ-9)**

Over the last two weeks how often have you been bothered by any of the following problems? (Please circle the number to indicate your answer)

|  | Not at all | Several days | More than half days | Nearly every day |
| --- | --- | --- | --- | --- |
| 601. Little interest or pleasure in doing things | 0 | 1 | 2 | 3 |
| 602. Feeling down, depressed or  hopeless | 0 | 1 | 2 | 3 |
| 1. Trouble failing or staying asleep or sleep too much | 0 | 1 | 2 | 3 |
| 1. Feeling of tired or having little energy. | 0 | 1 | 2 | 3 |
| 1. Poor appetite or over eating | 0 | 1 | 2 | 3 |
| 1. Feeling bad about yourself or that you are a failure or have let yourself or your family down | 0 | 1 | 2 | 3 |
| 1. Trouble concentrating on things as reading the newspaper or watching television | 0 | 1 | 2 | 3 |
| 1. Moving or speaking so slowly that other people could have noticed or the opposite being so fidgety or restless that you have been moving around a lot more than usual | 0 | 1 | 2 | 3 |
| 1. Thought that you would be better off dead or hurting your self | 0 | 1 | 2 | 3 |
| Total |  |  |  |  |

**Part 7: Psychosocial factors**

**7.1. Level of social supports;**

The following three questions ask about how you experience your social relationships. The inquiry is about your immediate personal experience. Please indicate the option that represents your experience.

| 701 | How many people are so close to you that you can count on them if you have serious personal problems (choose one option)? | None      1 | 1or 2      2 | 3-5    3 | More than 5    4 |  |
| --- | --- | --- | --- | --- | --- | --- |
| 702 | How much concern do people show in what you are doing (choose one option)? | A lot of concern and interest  5 | Some concern and interest  4 | Uncertain  3 | Little concern and interest  2 | No concern and interest  1 |
| 703 | How easy is it to get practical help from neighbors if you should need it (choose one option)? | Very difficult    1 | Difficult    2 | Possible    3 | Easy    4 | Very easy    5 |

**7.2. Perceived stress scale**

Circle the number that best describes how much the following problems have bothered you during the past month.

| Se.no | Questions | Never | Almost never | Some times | Fairly often |
| --- | --- | --- | --- | --- | --- |
| 801 | In the last month, how often have you been upset because of something that happened unexpectedly? | 0 | 1 | 2 | 3 |
| 802 | In the last month, how often have you felt that you were unable to control the important things in your life? | 0 | 1 | 2 | 3 |
| 803 | In the last month, how often have you felt nervous and stressed? | 0 | 1 | 2 | 3 |
| 804 | In the last month, how often have you felt confident about your ability to handle your personal problems? | 0 | 1 | 2 | 3 |
| 805 | In the last month, how often have you felt that things were going your way? | 0 | 1 | 2 | 3 |
| 806 | In the last month, how often have you found that you could not cope with all the things that you had to do? | 0 | 1 | 2 | 3 |
| 807 | In the last month, how often have you been able to control irritations in your life? | 0 | 1 | 2 | 3 |
| 808 | In the last month, how often have you felt that you were on top of things? | 0 | 1 | 2 | 3 |
| 809 | In the last month, how often have you been angered because of things that happened that were outside of your control? | 0 | 1 | 2 | 3 |
| 810 | In the last month, how often have you felt difficulties were piling up so high that you could not overcome them? | 0 | 1 | 2 | 3 |

**I have finished my questions thank you for your participation!!!**
