# Supplementary material for: Magnitude and correlates of alcohol use disorder in south Gondar zone, northwest Ethiopia: A community based cross-sectional study
Source: PLoS One. 2021 Sep 30;16(9):e0257804. doi: 10.1371/journal.pone.0257804 (PMC8483395; doi:10.1371/journal.pone.0257804)
Supplement: Attachment — Submitted filename: reviewed.docx [file pone.0257804.s002.docx]

Thanks for your invitation to review this study. Since evidences related to substance use disorders like alcohol are scared in low and middle-income countries like ours particularly in Amhara region. Thus, this study added a valuable contribution to the medical field. So, to make this useful for the general public the authors need to incorporate the comments mentioned below

**Language**

I advise extensive editing/proof-reading of the written English and re-drafting before the paper can be considered suitable for publication, b/se there are many odd phares and language errors.

**Introduction**

1. You put repeated sentence at your introduction and citing different references (reference number 6&7). For example, in recent years, high-income countries have increased their attention to alcohol-related harm by implementing governmental regulations to reduce alcohol consumption. In recent years, high-income countries have increased their attention to alcohol-related harm by implementing governmental regulations to reduce alcohol consumption.

**Methods and materials**

1. Better to say "study setting" rather than study settings

**Study participants**

1. Have you included participants with mental illness?
2. Under study setting and populations, it is good to mention the numbers and the type of health institutions in South Gondar Zone. In addition to this I strongly recommend you to include the type of mental health services delivered in those hospitals

**Measurements**

1. Why you preferred to measure alcohol used disorder by using CAGE other than AUDIT? More clarification is required.

**Data collection**

2. How study participants were reached e.g., door to door knocking/ or telephone interview?

Discussion

3. In paragraph 1 and 2 of your discussion were mentions Brazil twice in contradicting sentences " However, the finding of the current study was lower than a study conducted in Brazil 50% (15), and Conversely, the finding of this study was higher than the findings in USA 13.9% (13), India, 9.4% (41), Uganda 9.8% (20), Colombia 9% (42), Brazil, 18.4% (14) ...
4. On paragraph 4 of your discussion, References are particularly useful to substantiate statements which could be considered strengthen your justification e.g. "The possible reason might be due to lack of experience in social relationship, social and psychological support from their neighborhood and relatives can lead to use alcohol"
5. How did you manage the participant who are eligible for dependence treatment during the data collection time?
